# Phosphorescent soft salt for ratiometric and lifetime imaging of intracellular pH variations†
†Electronic supplementary information (ESI) available: UV-visible spectrum, photoluminescence spectrum, ^1^H NMR spectra, MS spectra and cell imaging experiment. See DOI: 10.1039/c5sc04624f


**DOI:** 10.1039/c5sc04624f

**Published:** 2016-02-04

**Authors:** Yun Ma, Hua Liang, Yi Zeng, Huiran Yang, Cheuk-Lam Ho, Wenjuan Xu, Qiang Zhao, Wei Huang, Wai-Yeung Wong

**Affiliations:** a Institute of Molecular Functional Materials , Department of Chemistry and Partner State Key Laboratory of Environmental and Biological Analysis , Hong Kong Baptist University , Waterloo Road , Hong Kong , P. R. China . Email: rwywong@hkbu.edu.hk ; Fax: +852 34117348 ; Tel: +852 34117074; b Key Laboratory for Organic Electronics & Information Displays (KLOEID) , Institute of Advanced Materials (IAM) , Jiangsu National Synergetic Innovation Center for Advanced Materials (SICAM) , Nanjing University of Posts and Telecommunications , Nanjing 210023 , P. R. China . Email: iamqzhao@njupt.edu.cn ; Fax: +86 25 85866396 ; Tel: +86 25 85866396; c Institute of Polymer Optoelectronic Materials and Devices , State Key Laboratory of Luminescent Materials and Devices , South China University of Technology , Guangzhou 510640 , P. R. China

## Abstract

In contrast to traditional short-lived fluorescent probes, long-lived phosphorescent probes based on transition-metal complexes can effectively eliminate unwanted background interference by using time-resolved luminescence imaging techniques, such as photoluminescence lifetime imaging microscopy. Hence, phosphorescent probes have become one of the most attractive candidates for investigating biological events in living systems. However, most of them are based on single emission intensity changes, which might be affected by a variety of intracellular environmental factors. Ratiometric measurement allows simultaneous recording of two separated wavelengths instead of measuring mere intensity changes and thus offers built-in correction for environmental effects. Herein, for the first time, a soft salt based phosphorescent probe has been developed for ratiometric and lifetime imaging of intracellular pH variations in real time. Specifically, a pH sensitive cationic complex (**C1**) and a pH insensitive anionic complex (**A1**) are directly connected through electrostatic interaction to form a soft salt based probe (**S1**), which exhibits a ratiometric phosphorescent response to pH with two well-resolved emission peaks separated by about 150 nm (from 475 to 625 nm). This novel probe was then successfully applied for ratiometric and lifetime imaging of intracellular pH variations. Moreover, quantitative measurements of intracellular pH fluctuations caused by oxidative stress have been performed for **S1** based on the pH-dependent calibration curve.

## Abstract 2

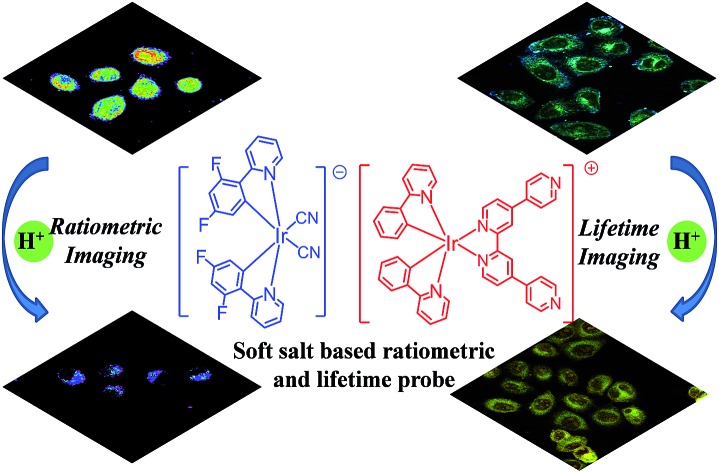
A novel soft salt based phosphorescent probe has been successfully developed for ratiometric and lifetime imaging of intracellular pH variations in real time.

## Introduction

Fluorescence bioimaging based on fluorescent probes provides a powerful approach for visualizing morphological details in biological systems with subcellular resolution.^1^ However, most traditional fluorescent probes often suffer from interference due to autofluorescence and scattered light, which increases background noise and reduces the signal-to-noise ratio (SNR). Recently, an emerging technique, namely, photoluminescence lifetime imaging microscopy (PLIM), has offered an effective way of eliminating unwanted background interference based on the lifetime difference between the probe and interference signal.^2^–^5^ In addition, lifetime as the detected signal is independent of excitation laser intensity, target molecule concentration and photobleaching, and is very beneficial for imaging applications. Phosphorescent transition-metal complexes (PTMCs), typically exhibiting long emission lifetime, large Stokes shift and high photostability,^6^–^11^ are ideal candidates for biological applications, especially for lifetime imaging applications,^12^–^21^ although examples of PTMCs for monitoring intracellular biomolecules by PLIM are quite rare.^12^–^19^

Despite the advantages of PTMCs-based probes, most previously reported probes were based on single emission intensity changes. The diversities in cell morphology within different districts might influence the quality and quantity of emission signals, which can result in substantial misinterpretations when dynamic changes of intracellular biomolecules are investigated. Therefore, accurate and quantitative measurements of the actual concentrations of intracellular biomolecules or the relative changes of concentrations in living cells are difficult. Ratiometric measurement is normally used to address this issue. It can permit simultaneous recording of the relative changes of two separated wavelengths instead of measuring single emission intensity changes and thus offers built-in correction for environmental effects, leading to a more favorable system for imaging living cells and tissues.^22^–^25^ However, most ratiometric probes developed recently for imaging of biological molecules are based on organic dyes or nanoparticles,^26^–^31^ only a few ratiometric PTMCs probes have been reported.^32^–^34^ It is still a challenge to design PTMCs-based ratiometric probes due to their complex excited-state properties.^35^–^38^

Luminescent ion pairs, which consist of two photoactive coordination complexes with opposite charges, are called “soft salts” due to the soft nature of the ions.^39^–^43^ Recently, Thompson and co-workers have studied the photophysical properties of soft salts in detail and successfully applied them in organic light-emitting diodes.^39^ However, extensive studies on soft salts have not received much attention yet. Considering that two emission wavelengths from a soft salt can be easily separated by chemical modification of cyclometalated ligands of the two ionic complexes, soft salts will be a good and versatile platform for the design of phosphorescent ratiometric probes. To date, however, the applications of soft salts in chemical sensing and biological systems are still unexploited areas.

Here we present the first example of a soft salt based ratiometric probe for imaging and measuring pH variations in living cells. Intracellular pH is a crucial parameter associated with cellular behaviors and pathological conditions, such as cell proliferation, apoptosis,^44^ drug resistance,^45^ enzymatic activity,^46^ and ion transport.^47^ Abnormal cellular pH value is an indicator of inappropriate cellular functions, which are associated with many common diseases, for example, stroke,^48^ cancer,^49^ and Alzheimer's disease.^50^ It is thus vital to monitor pH alterations in biological cells and tissues to understand physiological and pathological processes.^51^,^52^

**Fig. 1:**
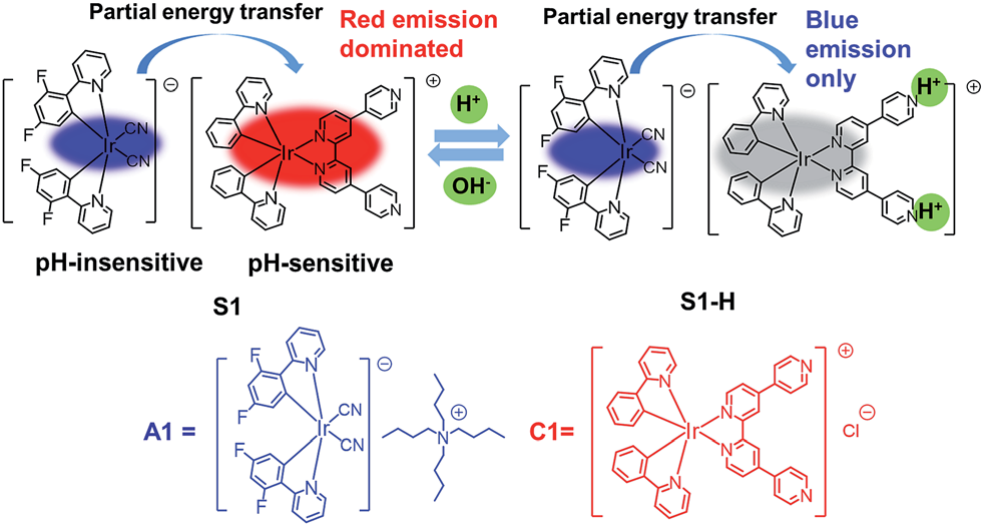
Design concept of a ratiometric pH probe and chemical structures of complexes **A1**, **C1** and **S1**.

Fig. 1 schematically describes the design concept. We selected the cationic complex **C1** with pendant pyridyl moieties as a pH-sensitive phosphor and the anionic complex **A1** as a pH-insensitive phosphor. These two luminophores are connected by electrostatic interaction to form the soft salt complex **S1**. Complex **S1** is expected to give two emission bands, namely pH-insensitive blue and pH-sensitive red phosphorescence emissions. Thus, the ratio of the phosphorescence intensities can respond to different pH values (2.03–7.94). Moreover, **S1** exhibits two well-resolved emission peaks separated by about 150 nm (from 475 to 625 nm), which avoids mutual interference of two emission bands and allows for high-resolution and sensitive ratiometric response of pH variations. Hence, complex **S1** could act as an ideal ratiometric probe to monitor pH variations in biological cells. Furthermore, to utilize the long phosphorescence lifetime of complex **S1**, PLIM experiments were carried out to monitor intracellular pH alterations.

## Results and discussion

### Synthetic procedures

The cyclometalated iridium(iii) chloro-bridged dimer [Ir(C^N)_2_Cl]_2_ (C^N = 2-(2,4-difluorophenyl)pyridine (dfppy) or 2-phenylpyridine (ppy)) and the 2,2′:4,4′′:4′,4′′′-quaterpyridyl (qpy) ligand were synthesized according to the literature methods.^53^,^54^ The cationic iridium(iii) complex [Ir(ppy)_2_qpy]^+^Cl^–^ (**C1**) was prepared by refluxing bis(cyclometalated) iridium(iii) dichloro-bridged dimer in the presence of an excess of qpy ligand.^55^ The anionic iridium(iii) complex [Ir(dfppy)_2_(CN)_2_]^–^Bu_4_N^+^ (**A1**) was synthesized from [Ir(dfppy)_2_Cl]_2_ and tetrabutylammonium cyanide (10 equiv.) in dichloromethane at 50 °C for 4 h. By mixing two oppositely charged iridium(iii) complexes **A1** and **C1** (1 : 1 molar ratio) in a mixture of CH_3_CN–H_2_O (1 : 1, v/v) at room temperature, the soft salt (**S1**) was obtained through the metathesis reaction. The above complexes were characterized by ^1^H and ^13^C NMR spectroscopy, MALDI-TOF spectrometry and elemental analysis.

### Photophysical properties

**Fig. 2:**
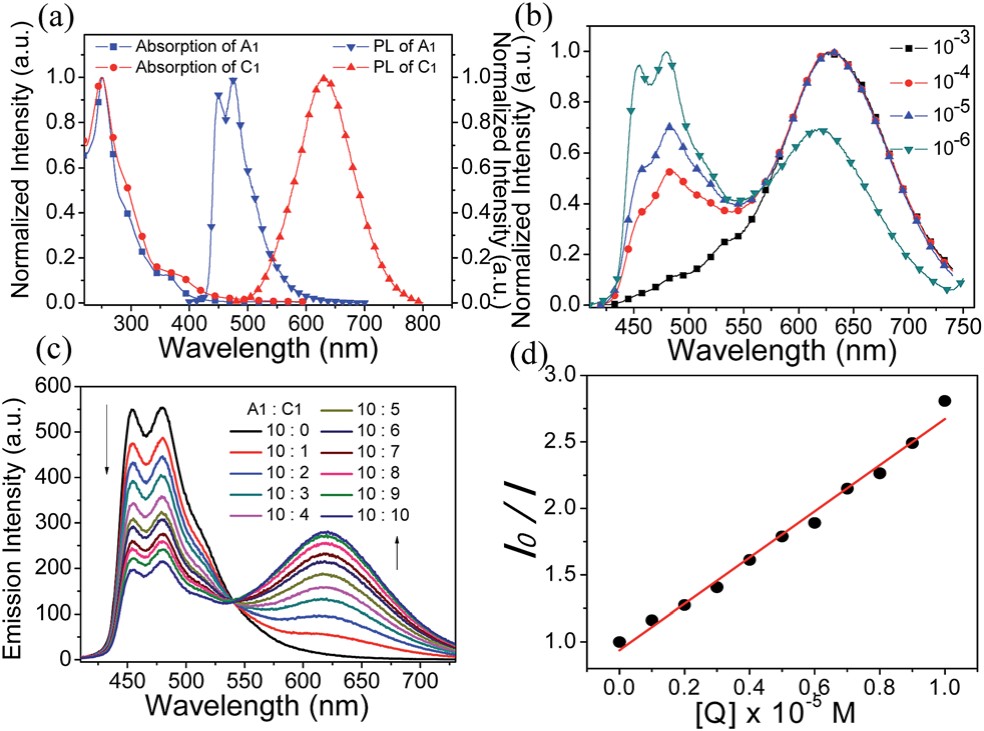
(a) Normalized absorption and photoluminescence spectra of **A1** and **C1** in acetonitrile solution. (b) Photoluminescence spectra of **S1** at different concentrations in acetonitrile solution. (c) Photoluminescence spectra of anionic complex **A1** (10^–5^ M) in acetonitrile solution with various amounts of cationic complex **C1** (0–1.0 × 10^–5^ M). (d) Stern–Volmer plot of the quenching study between **C1** and **A1** ([Q] is the concentration of quencher).

The photophysical data of **A1**, **C1** and **S1** are summarized in Table S1 (ESI†). Spectroscopic results of **A1** and **C1** in acetonitrile are shown in Fig. 2a. The emission peaks of anionic complex **A1** at around 451 and 475 nm display vibronic progressions, which is a result of the triplet ligand-centered (^3^LC) transition on the cyclometalated ligands.^56^ The cationic complex **C1** shows a broad and featureless spectrum with the emission maximum at 625 nm. Therefore, **C1** is expected to emit from a metal-to-ligand charge-transfer (MLCT) state.^57^ The photoluminescence (PL) spectrum of **S1** exhibits concentration dependence (Fig. 2b). The intensity ratio of emission peaks of **A1** and **C1** varies significantly depending on the solution concentration, suggesting that the degree of energy transfer between the two complexes is different. At 380 nm excitation, the emission is mainly from the anionic complex **A1** at a relatively low concentration of 10^–6^ M, which might be due to the fact that the quantum efficiency of **A1** is much higher than that of **C1**. The blue emission decreases as the concentration of solution increases, with the cationic complex **C1** acting as a quencher of **A1**, and the emission of **A1** is barely observed at a concentration of 10^–3^ M or above.

The quenching study was carried out to investigate the energy transfer between the two ionic components in **S1**. As shown in Fig. 2c, with the addition of an increasing amount of cationic complex **C1** into an acetonitrile solution of the anionic complex **A1** (10^–5^ M), the red emission increased gradually at the expense of the blue emission from **A1**. This quenching effect can be attributed to the intermolecular triplet–triplet energy transfer.^58^–^60^ The results indicate that the energy transfer/quenching process is very efficient between the two ionic complexes. The quenching rate constant (*K*_q_) can be derived by dividing the slope of the fitted straight line by *I*_0_ (the luminescence intensity with no quencher present) (Fig. 2d). The calculation yielded a *K*_q_ value of 5.49 × 10^10^ M^–1^ s^–1^.

### Ratiometric response to pH variations

**Fig. 3:**
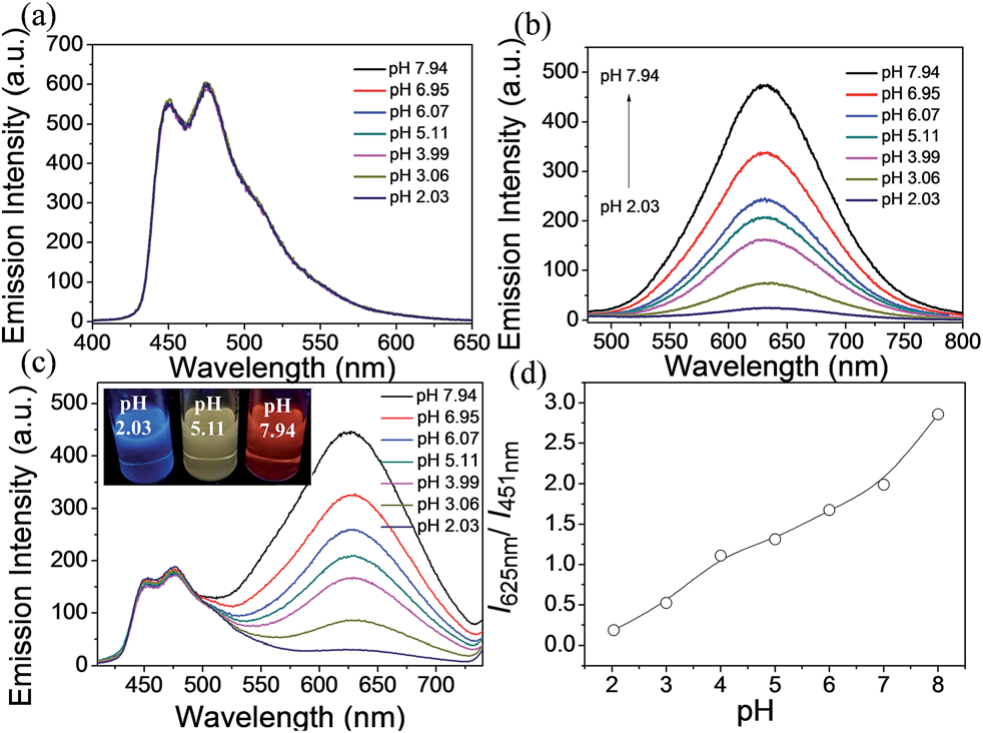
(a) Changes in the phosphorescence emission spectra of **A1** (2.0 × 10^–5^ M) in the pH range of 2.03–7.94 in CH_3_CN/buffer (1 : 9, v/v). (b) Changes in the phosphorescence emission spectra of **C1** (2.0 × 10^–5^ M) in the pH range of 2.03–7.94 in CH_3_CN/buffer (1 : 9, v/v). (c) Changes in the phosphorescence emission spectra of **S1** (2.0 × 10^–5^ M) in the pH range of 2.03–7.94 in CH_3_CN/buffer (1 : 9, v/v). (d) Plot of *I*_625 nm_/*I*_451 nm_*versus* pH values. *I*_625 nm_ and *I*_451 nm_ indicate the phosphorescence intensity at 625 nm and at 451 nm, respectively.

The phosphorescence emission spectra of **A1**, **C1** and **S1** were examined in acetonitrile/buffer (1 : 9, v/v) at various pH values (2.03–7.94). There are no obvious spectral variations for **A1** solutions of different pH values (Fig. 3a). For **C1**, the emission intensity at 625 nm decreases dramatically with the decrease in pH value, which can serve as an on–off single intensity based pH probe (Fig. 3b). Protonation makes the pyridine ring in the ancillary ligand of **C1** a stronger electron acceptor, which can cause quenching of the phosphorescence. To realize the ratiometric probe, a soft salt **S1** constituted of **A1** and **C1** by electrostatic interaction has been developed. The phosphorescence spectral changes of **S1** at different pH values are displayed in Fig. 3c. Increasing the pH value results in a higher phosphorescence intensity of **C1** at 625 nm (*I*_625 nm_), while the emission intensity of **A1** at 451 nm (*I*_451 nm_) remains unchanged. Such a change in phosphorescence emission color from blue to red with increasing pH value can be easily observed by the naked eye (Fig. 3c). The relative ratio of phosphorescence intensities (*I*_625 nm_/*I*_451 nm_) increased by 16-fold (from 0.18 to 2.86) over the pH range of 2.03–7.94 (Fig. 3d), which covers most physiological pH values. In addition, the phosphorescence response of **S1** to pH value displays an excellent reversibility (Fig. S3†).

We investigated the interference to the pH measurement by biological molecules, and the phosphorescence spectral responses of **S1** in the presence of oxidative-stress-associated redox chemicals (such as cysteine (Cys), homocysteine (Hcy), glutathione (GSH) and H_2_O_2_) and essential metal ions (such as K^+^, Na^+^, Zn^2+^, Cu^2+^, Ca^2+^, Mn^2+^, Mg^2+^, Fe^2+^ and Fe^3+^) were measured. There are no apparent spectroscopic changes detected (Fig. S4†), which indicates that **S1** could act as a phosphorescent probe for the detection of intracellular pH alterations without any interference. In addition, the stability of **S1** in acetonitrile/buffer (1 : 9, v/v) at 37 °C was investigated. Fig. S5† shows that the relative ratio of phosphorescence intensities (*I*_625 nm_/*I*_451 nm_) barely changed at 37 °C even after 2 h, indicating the good stability of **S1**.

### Cytotoxicity

The cytotoxicity towards HepG-2 cells was evaluated by the standard MTT (MTT = 3-(4,5-dimethylthiazol-2-yl)-2,5-diphenyl-tetrazolium bromide) assay. The results are illustrated in Fig. S6.† After treatment of living HepG-2 cells with different concentrations of **S1** for 24 h, the cellular viabilities were estimated to be approximately 85% at 200 μM, apparently indicating good biocompatibility and low cytotoxicity of **S1**.

### Ratiometric imaging

Practical application of complexes **C1**, **A1** and **S1** in luminescence imaging of living HepG-2 cells was investigated using confocal luminescence microscopy. After incubation with 10 μM of **C1** (**A1** or **S1**) for 1 h at 37 °C, notable intracellular luminescence was observed in HepG-2 cells (Fig. S7†). The overlay of confocal luminescence and bright-field images demonstrated that luminescence was evident in the cytoplasm region. To determine the kinetics of complex internalization, time-lapse imaging was carried out to monitor the progression of **A1**, **C1** and **S1** in HepG-2 cells *via* a Live Cell Workstation. Confocal images were obtained after 15 min, 30 min, 45 min and 1 h, respectively. As shown in Fig. S8,† only very weak luminescence was observed in the cells in the first 45 min for **A1**, indicating a slow cellular uptake rate of **A1**. In contrast, for **C1**, notable luminescence was detected in the cells within a short period of time (15 min).

**Fig. 4:**
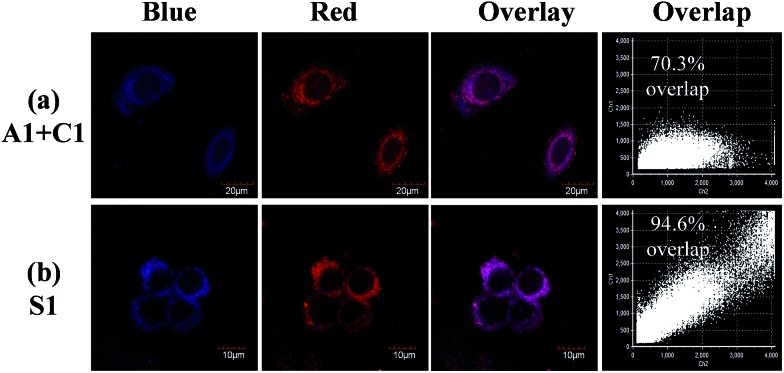
(a) Living HepG-2 cells co-stained with 10 μM **A1** and **C1** for 1 h at 37 °C, and (b) living HepG-2 cells incubated with **S1** under the same conditions.

These findings suggest that the cellular uptake rates are different for **A1** and **C1**, which might be due to the different ionic natures of the complexes. However, when **S1** was treated with HepG-2 cells, both the blue and red luminescence were observed in the cells at 15 min (Fig. S9†), implying that the cellular uptake rate for each counterpart of the soft salt is similar. In addition, the overlapping rate was calculated, which showed that the two counterparts of the soft salt remained intact in the cells. From Fig. 4, we can see that the overlapping rate between the blue and red channels was calculated to be 70.3% when the living cells were co-stained with **A1** and **C1**. However, after the treatment of living cells with **S1**, the overlapping rate of the two channels was 94.6%. These observations suggest that the cationic and anionic parts of **S1** remain intact rather than fall apart in the cells.

**Fig. 5:**
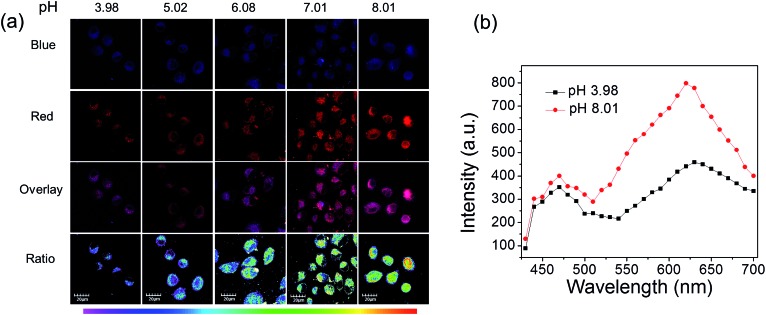
(a) Phosphorescence images of **S1** in HepG-2 cells clamped at pH 3.98, 5.02, 6.08, 7.01 and 8.01, respectively. The excitation wavelength was 405 nm and the images of the first row (blue channel) and second row (red channel) were collected in the ranges of 430–480 nm and 600–700 nm, respectively. Overlay images (third row) and ratio images obtained from the red and blue channels (fourth row). (b) Phosphorescence emission spectra of the HepG-2 cells at pH 3.98 and 8.01.

Next, these complexes were used for monitoring intracellular pH changes. The HepG-2 cells were cultured with 10 μL nigericin (10 ng mL^–1^) for 10 min to homogenize the intracellular pH value first. Remarkable intracellular luminescence enhancement was observed with the increase in pH value for **C1** (Fig. S10 and S11†). In contrast, no obvious luminescence intensity change can be detected in HepG-2 cells for **A1** (Fig. S10 and S11†). We subsequently exploited the ratiometric probe **S1** to examine the change in the cellular pH value in living cells. As shown in Fig. 5a, the luminescence from the red channel (600–700 nm) in cells increases with increasing pH value, whereas that from the blue channel (430–480 nm) hardly alters. These imaging results have further been demonstrated by phosphorescence emission spectra of HepG-2 cells at pH 3.98 and 8.01 (Fig. 5b), which show significant variation in the red channel but small change in the blue channel. The obtained intracellular phosphorescence emission spectra are also similar to those measured in solution. Thus, the variations in the ratio of blue to red intensity reveal the ability of **S1** to measure a pH-dependent signal linearly over the pH range of 4–8 (*R*^2^ = 0.9854; Fig. 7a). In addition, it is important to understand how **S1** behaves at different concentrations inside the cells because **S1** shows concentration-sensitive emission. Therefore, living cells were incubated with 1 μM **S1** to detect the intracellular pH variations. As shown in Fig. S12,† the obtained imaging results are similar to those measured at the concentration of 10 μM.

### Lifetime imaging

**Fig. 6:**
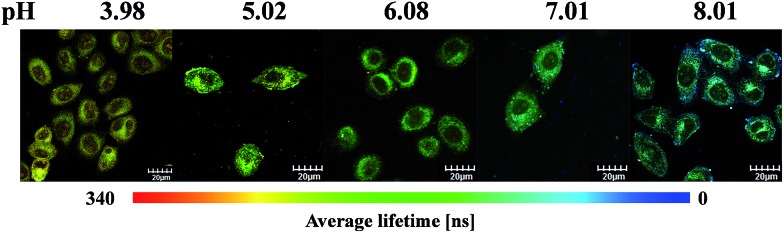
Phosphorescence lifetime images of **S1** in living HepG-2 cells at different pH values. HepG-2 cells were incubated for 1 h at 37 °C.

To utilize the long phosphorescence lifetime of complex **S1**, a PLIM experiment was carried out for living HepG-2 cells. We expect that PLIM can separate the long-lived phosphorescence signal from other contributions to the total photoluminescence. The emission lifetimes of **S1** were first measured in CH_3_CN/buffer (1 : 9, v/v) of different pH values (Table 1). The lifetime from 625 nm is increased with increasing pH value (from 73 to 328 ns), but that from 451 nm stays unchanged (around 700.0 ns), which is similar to the variation trend of the emission intensity. Then, the PLIM experiment was performed after the living HepG-2 cells were incubated with 10 μM **S1** at 37 °C for 1 h. As shown in Fig. 6, the average photoluminescence lifetime (*τ*_avg_) of **S1** experienced an obvious increase with the decrease in pH value. The *τ*_avg_ of **S1** was determined to be 178 ± 3.2 ns by PLIM when the intracellular pH value was 8.01. As the intracellular pH value decreased from 8.01 to 3.98, the *τ*_avg_ of **S1** was measured to be 209 ± 1.9 ns, 247 ± 2.2 ns, 271 ± 2.3 ns and 312 ± 3.1 ns, respectively (Fig. S14†). The *τ* averages of **S1** from cell growth media at varying pH values were collected by confocal TCSPC-PLIM (TCSPC = time-correlated single photon counting) to study how well the *τ* average from cells matched with the solution data at varying pH value (Fig. S16†). From Fig. S17† we can see that the variation trend of emission lifetime of **S1** is similar in the solution and cells. However, it is found that the emission lifetimes in the solution are shorter than those in the cells. This might be because the oxygen content inside cancer cells is lower than that in air-equilibrated solution. The PLIM experiment collected photons randomly from both blue and red emissions, and only the red emission was affected by the change in pH value. Therefore, more photons from the blue emission are collected as the pH decreases due to the reduction in the red emission intensity. And the fact that *τ*_625 nm_ is decreasing as the pH decreases (Table 1) indicates that the increase in average photoluminescence lifetime from pH 8 (*τ*_avg_ = 178 ± 3.2 ns) to pH 4 (*τ*_avg_ = 312 ± 3.1 ns) is reasonable. In addition, *τ*_avg_ of **S1** (1 μM) is consistent with the results obtained at the concentration of 10 μM (Table 2, Fig. S18 and S19†). Thus, the result has demonstrated that **S1** has the ability to detect the intracellular pH alterations by the photoluminescence lifetime, which highlights the capability of removing the background fluorescence.

**Table 1:** Emission lifetimes of **S1** at different pH values

	pH 3.99	pH 5.11	pH 6.07	pH 6.95	pH 7.94
*τ*_451 nm_ (ns)	705 ± 1.6	697 ± 1.4	739 ± 1.2	725 ± 1.1	711 ± 0.9
*τ*_625 nm_ (ns)	73 ± 3.2	131 ± 1.5	169 ± 1.7	184 ± 1.6	328 ± 1.7

**Table 2:** Emission lifetimes of **S1** at different pH values *via* TCSPC-PLIM

	pH 3.98	pH 5.02	pH 6.08	pH 7.01	pH 8.01
RPMI (ns)	261 ± 1.4	238 ± 1.3	211 ± 1.7	176 ± 1.4	141 ± 2.8
Cells (ns)	312 ± 3.1	271 ± 2.3	247 ± 2.2	209 ± 1.9	178 ± 3.2

### Quantitative measurement of intracellular pH fluctuations

**Fig. 7:**
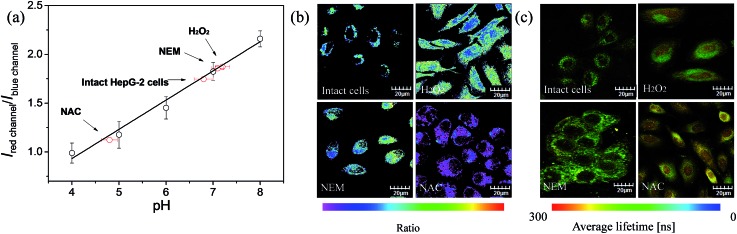
(a) Intracellular pH calibration curve of **S1** in HepG-2 cells. (b) Ratiometric images and (c) phosphorescence lifetime images of **S1** (10 μM). Intact cells, H_2_O_2_ (100 μM) treated, NEM (100 μM) treated and NAC (100 μM) treated cells were incubated for 1 h at 37 °C.

The ratio channel, obtained based on the above two distinguishable emission channels, shows a characteristic pH-dependent signal, demonstrating the ability of **S1** to examine a pH-dependent signal linearly over the pH range of 3.98 to 8.01 (Fig. 7a). According to this calibration curve, the averaged intracellular pH value of intact HepG-2 cells was measured to be 6.80 ± 0.20 (Fig. 7). Furthermore, the effects of different redox substances on intracellular pH fluctuations were investigated based on the calibration curve. As shown in Fig. 7a and b, the pH value for H_2_O_2_ treated cells was determined to be 7.20 ± 0.15, which indicates that H_2_O_2_ makes the HepG-2 cells more basic. This observation is in good agreement with the previous report that oxidative stress (such as H_2_O_2_) can cause inactivation of lysosomal V-ATPase, consequently resulting in increasing the pH value of lysosomes.^61^ Then, NEM (*N*-ethylmaleimide, a GSH inhibitor) or NAC (*N*-acetylcysteine, a GSH precursor) was applied to HepG-2 cells to control the intracellular GSH level. The intracellular pH value was measured to be 7.10 ± 0.12 after decreasing the concentration of GSH by NEM (Fig. 7). Possible reasons for the basification of cells are as follows: (i) an oxidative cellular environment caused by the decrease of GSH induces lysosomal inactivation; (ii) the function of the Na^+^/H^+^ antiporter may be affected by the decrease of GSH level.^62^ Interestingly, the generation of GSH by NAC decreases the intracellular pH to 4.80 ± 0.16 (Fig. 7). We believe that a reductive cellular environment caused by the high concentration of GSH induces the activation of lysosomal V-ATPase, which is a possible explanation for this acidification.^63^ Ratiometric images directly reveal the intracellular pH changes caused by the oxidative stress (Fig. 7b). To take advantage of ratiometric measurement, accurate and quantitative determinations of the actual intracellular pH value and its relative changes can be well achieved. Moreover, when the lifetime serves as a signal, these intracellular pH variations could also be detected by PLIM. Fig. 7c shows the phosphorescence lifetime images of intact cells, H_2_O_2_ treated, NEM treated and NAC treated cells, and their *τ*_avg_ were determined to be 217 ns, 206 ns, 201 ns and 263 ns, respectively. This result highlights that the detection of intracellular pH alterations by the lifetime signal can avoid the interferences from autofluorescence, scattered light as well as other technical artifacts.

## Conclusions

In conclusion, we have developed a novel soft salt based phosphorescent probe. This type of probe consists of two oppositely charged ionic complexes with two distinguishable emission colors, which makes it a perfect candidate as a ratiometric probe. The emission color of **S1** changes from blue to red with increasing pH value. **S1** is cell-permeable and exhibits low cytotoxicity, and it has been successfully applied for ratiometric pH imaging with the use of confocal microscopy, demonstrating its great potential for intracellular environmental monitoring. Furthermore, phosphorescence lifetime imaging experiments can detect intracellular pH variations by photoluminescence lifetime measurements, which allowed for eliminating background fluorescence and selecting long-lived phosphorescence images. Quantitative measurement of intracellular pH fluctuations caused by oxidative stress has been successfully carried out for **S1** based on the pH-dependent calibration curve. To our knowledge, this work represents the first example of a soft salt based probe for chemical sensing and biological applications. We expect that this work can provide valuable information for the future rational design of phosphorescent ratiometric and lifetime probes.

## Experimental

### Materials and general experiments

Commercially available chemical reagents were used without further purification. All solvents were purified before use. The solvents were carefully dried and distilled from appropriate drying agents prior to use. ^1^H and ^13^C NMR spectra were recorded with a Bruker Ultrashield 400 MHz FT-NMR spectrometer. Mass spectra were obtained with a Bruker Autoflex matrix assisted laser desorption/ionization time of flight mass spectrometer (MALDI-TOF MS). UV-visible absorption spectra were recorded with a HP UV-8453 spectrophotometer. Photoluminescence spectra were measured with an Edinburgh Instrument FLS920 combined fluorescence lifetime and steady state spectrophotometer that was equipped with a red-sensitive single-photon counting photomultiplier in Peltier Cooled Housing. The quantum efficiencies of complexes were measured in solutions at room temperature with an aerated aqueous solution of [Ru(bpy)_3_]Cl_2_ as an external standard (*Φ* = 0.028).

### Synthesis of 2,2′:4,4′′:4′,4′′′-quaterpyridyl (qpy)

4,4′-Bipyridine (2 g) and 10% palladium on carbon (400 mg) in DMF were heated at 180 °C for 48 h under an inert atmosphere of nitrogen. After being cooled to room temperature, the mixture was filtered and DMF was removed under reduced pressure. The residual solid was recrystallized from acetone to give white crystals. Yield 19%. ^1^H NMR (400 MHz, CDCl_3_): *δ* (ppm) 8.81 (d, *J* = 8 Hz, 2H), 8.76 (d, *J* = 8 Hz, 6H), 7.67 (dd, *J* = 4 Hz, 4H), 7.59 (dd, *J* = 8 Hz, 2H). MS (MALDI-TOF) [*m*/*z*]: 310.4 [M^+^].

### Synthesis of **C1**

A mixture of iridium(iii) bis(2-phenylpyridine) dichloro-bridged dimer (34.5 mg, 0.032 mmol) and 2,2′:4,4′′:4′,4′′′-quaterpyridyl (25 mg, 0.08 mmol) was dissolved in a mixture of dichloromethane and methanol (2 : 1, v/v) and the mixture was refluxed for 16 h. The solution was concentrated and washed with hexane to afford the crude product. Afterwards, the product was recrystallized by vapor diffusion of diethyl ether into acetonitrile. Yield 65%. ^1^H NMR (400 MHz, acetonitrile-*d*_3_): *δ* (ppm) 8.97 (s, 2H), 8.82 (d, *J* = 8 Hz, 4H), 8.10 (d, *J* = 4 Hz, 4H), 7.87–8.81 (m, 10H), 7.70 (d, *J* = 4 Hz, 2H), 7.09 (dd, *J* = 16 Hz, 4H), 6.98 (t, *J* = 16 Hz, 2H), 6.33 (d, *J* = 8 Hz, 2H). ^13^C NMR (100 MHz, acetone-*d*_6_), *δ* (ppm): 168.67, 157.71, 152.21, 151.90, 151.23, 150.28, 149.41, 144.96, 143.86, 139.74, 132.50, 131.40, 127.37, 125.95, 124.61, 123.98, 123.58, 122.48, 120.96. MS (MALDI-TOF) [*m*/*z*]: 811.8 [M – Cl]^+^. Elemental analysis (calcd, found for C_40_H_48_F_4_IrN_5_): C (55.41, 55.74), H (5.58, 5.93), N (8.08, 8.24).

### Synthesis of **A1**

Iridium(iii) bis(2-(2,4-difluorophenyl)pyridine) dichloro-bridged dimer (146 mg, 0.12 mmol) was combined with tetrabutylammonium cyanide (360 mg, 1.2 mmol) in dichloromethane at 50 °C for 4 h. After removing dichloromethane under reduced pressure, the product was purified by aluminum oxide (chromatography) with dichloromethane and methanol (10 : 1, v/v) as the eluent. Yield 78%. ^1^H NMR (400 MHz, DMSO-*d*_6_): *δ* (ppm) 9.53 (d, *J* = 8 Hz, 2H), 8.20 (d, *J* = 8 Hz, 2H), 8.02 (t, *J* = 16 Hz, 2H), 7.44 (t, *J* = 12 Hz, 2H), 6.64–6.59 (m, 12H), 5.52 (d, *J* = 8 Hz, 2H), 3.33–3.17 (m, 8H), 1.61–1.53 (m, 8H), 1.34–1.25 (m, 8H), 0.92 (t, *J* = 16 Hz, 12H). ^13^C NMR (100 MHz, DMSO-*d*_6_): *δ* (ppm) 170.2, 163.42, 154.97, 145.83, 139.76, 137.44, 132.17, 129.64, 124.72, 123.36, 121.62, 120.11, 59.49, 31.15, 23.52, 19.67, 13.95. MS (MALDI-TOF) [*m*/*z*]: 625.1 [M – Bu_4_N]^–^. Elemental analysis (calcd, found for C_42_H_30_ClIrN_6_): C (59.60, 59.95), H (3.57, 3.89), N (9.93, 10.11).

### Synthesis of **S1**

[Ir(dfppy)_2_(CN)_2_]^–^Bu_4_N^+^ (25 mg, 0.035 mmol) and [Ir(ppy)_2_qpy]^+^Cl^–^ (25 mg, 0.04 mmol) were added to CH_3_CN (10 mL). The reaction mixture was stirred for 2 h at room temperature and then extracted with CH_2_Cl_2_. The solution was washed with water several times to remove the counterions and concentrated by rotary evaporation. The resulting solid was washed with diethyl ether to afford **S1** as a red solid. Yield 85%. ^1^H NMR (400 MHz, acetonitrile-*d*_3_): *δ* (ppm) 9.51 (d, *J* = 4 Hz, 2H), 8.93 (s, 2H), 8.71 (d, *J* = 4 Hz, 4H), 8.15 (d, *J* = 4 Hz, 2H), 8.02 (d, *J* = 8 Hz, 4H), 7.81–7.74 (m, 12H), 7.62 (d, *J* = 4 Hz, 2H), 7.17 (t, *J* = 12 Hz, 2H), 7.02–6.95 (m, 4H), 6.89 (t, *J* = 12 Hz, 2H), 6.33 (t, *J* = 16 Hz, 2H), 6.25 (d, *J* = 8 Hz, 2H), 5.55 (d, *J* = 8 Hz, 2H). ^13^C NMR (100 MHz, DMSO-*d*_6_): *δ* (ppm) 167.22, 164.30, 156.61, 156.36, 154.18, 150.94, 149.68, 147.96, 146.30, 144.88, 144.30, 142.94, 139.37, 138.04, 131.52, 130.80, 128.59, 128.27, 126.89, 125.64, 124.51, 123.86, 123.54, 123.02, 122.82, 122.52, 122.30, 121.88, 120.60, 118.50, 112.30, 97.03, 55.38. MS (MALDI-TOF) [*m*/*z*]: 811.8 [M^+^], 625.1 [M^–^]. Elemental analysis (calcd, found for C_66_H_42_F_4_Ir_2_N_10_): C (55.22, 55.58), H (2.95, 3.38), N (9.76, 9.69).

### Cell culture

The HepG-2 cell line was supplied by the Institute of Biochemistry and Cell Biology, SIBS, CAS (China). The HepG-2 cells were grown in RPMI 1640 (Roswell Park Memorial Institute's Medium) supplemented with 10% FBS (Fetal Bovine Serum) at 37 °C and 5% CO_2_. Cells (5 × 10^8^ per L) were plated on 18 mm glass coverslips and allowed to adhere for 24 h.

### Cytotoxicity assay

The cytotoxicity of the complexes toward the HepG-2 cells was measured by the methyl thiazolyl tetrazolium assay. Before incubation at 37 °C under 5% CO_2_ atmosphere for 24 h, HepG-2 cells in log phase were seeded into a 96-well cell-culture plate at 1 × 10^4^ per well. The **S1** solution (100 μL per well) at concentrations of 200, 100, 50 and 25 μM was added to the wells of the treatment group, and MTT containing 0.2% DMSO (100 μL per well) to the negative control group. The cells were incubated at 37 °C under 5% CO_2_ atmosphere for 24 h. 20 μL MTT solution (5 mg mL^–1^) was added to each well of the 96-well assay plate, and the solution was incubated for another 3 h under the same conditions. A Tecan Infinite M200 monochromator based multifunction microplate reader was used for measuring the OD570 (absorbance value) of each well referenced at 690 nm. The following formula was used to calculate the viability of cell growth:Viability (%) = [(mean absorbance value of treatment group)/(mean absorbance value of control)] × 100.

### Cell imaging

**C1**, **A1** and **S1** were dissolved in DMSO/RPMI 1640 (v/v, 1 : 99) to yield 10 μM solutions. Before washing with PBS, the HepG-2 cells were incubated solely with the solution of **C1** (or **A1** and **S1**) for 1 h at 37 °C. Then, the experiments were carried out on an Olympus FV1000 laser scanning confocal microscope and a 60× oil-immersion objective lens. A semiconductor laser served as the excitation source of the HepG-2 cells incubated with **C1** (or **A1** and **S1**) at 405 nm. The overlay images were generated by the Fluoview Viewer software. The ratio images were analyzed with Kodak Molecular Imaging Software, and the ratio of region of interest (ROI) was calculated pixel-by-pixel. All data were expressed as mean ± standard deviation.

### PLIM imaging

The PLIM image setup is integrated with an Olympus IX81 laser scanning confocal microscope. The fluorescence signal was detected by the system of the confocal microscope and correlative calculation of the data was carried out by professional software which was provided by Pico Quant Company. The light from the pulse diode laser head (Pico Quant, PDL 800-D) with excitation wavelength of 405 nm and frequency of 1 MHz (>1 μs) was focused onto the sample with a 40×/NA 0.95 objective lens for single-photon excitation. The PLIM data were processed in SymPhoTime 64 pro software (Pico Quant Company), and exported in ASCII (line profiles) or BMP (images) format. PLIM data obtained from 256 × 256 regions of interest were fit using double exponential tailfit, binning factor 1 in SymPhoTime 64 pro software. Fit curves in each pixel, excluding dark regions, yielded a lifetime distribution over the whole image, with a lifetime being displayed on the *x*-axis and the abundance of each lifetime on the *y*-axis. The average lifetime (50% of the total integral is reached) and half-width (difference between lifetimes at which half-maximal abundance is reached) were calculated from the distribution curve.

### Intracellular pH calibration

The HepG-2 cells were incubated at 37 °C for 10 min in high K^+^ buffer (30 mM NaCl, 120 mM KCl, 1 mM CaCl_2_, 0.5 mM MgSO_4_, 1 mM NaH_2_PO_4_, 5 mM glucose, 20 mM HEPES) with various pH values (3.98–8.01) in the presence of 10 μL nigericin (10 ng mL^–1^). HepG-2 cells were incubated with **C1** (or **A1** and **S1**) for 1 h at 37 °C. The phosphorescence and lifetime images were then measured, and the pH calibration curve was constructed with an Olympus FV1000 confocal microscope.

## Supplementary Material

Supplementary information
