# Supplementary material for: Phosphorescent soft salt for ratiometric and lifetime imaging of intracellular pH variations
Source: Chem Sci. 2016 Feb 4;7(5):3338–46. doi: 10.1039/c5sc04624f (PMC6006953; doi:10.1039/c5sc04624f)
Supplement: Supplementary file 1 [file SC-007-C5SC04624F-s001.pdf]

Electronic Supplementary Material (ESI) for Chemical Science.
This journal is © The Royal Society of Chemistry 2016

# *Electronic Supplementary Information*

Phosphorescent soft salt for ratiometric and lifetime imaging of intracellular pH variations

Yun Ma,[a] Hua Liang,[b] Yi Zeng,[a] Huiran Yang,[b] Cheuk-Lam Ho,[a] Wenjuan, Xu,[b] Qiang Zhao,*[b] Wei Huang,[b] and Wai-Yeung Wong*[ac]

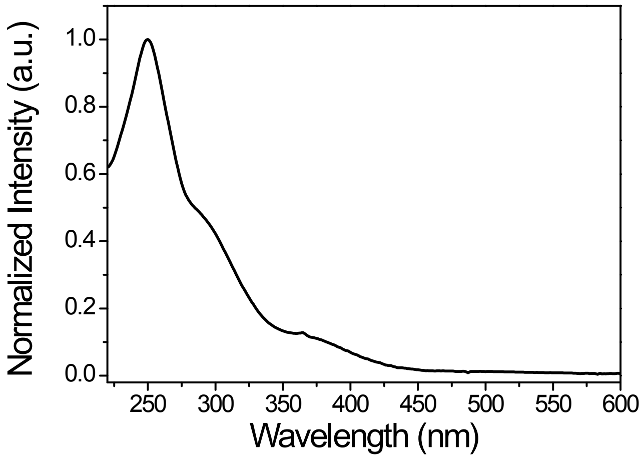


**Fig. S1** Normalized absorption spectrum of **S1** in acetonitrile solution.

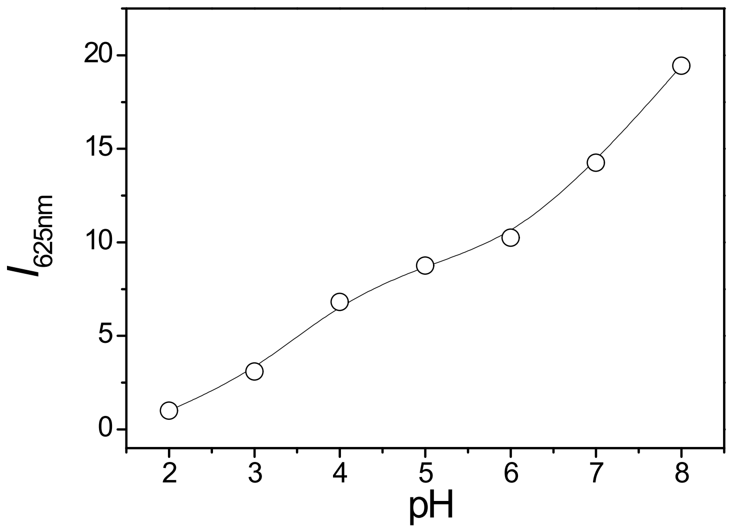


**Fig. S2** Plot of $I_{625nm}$ versus pH value.

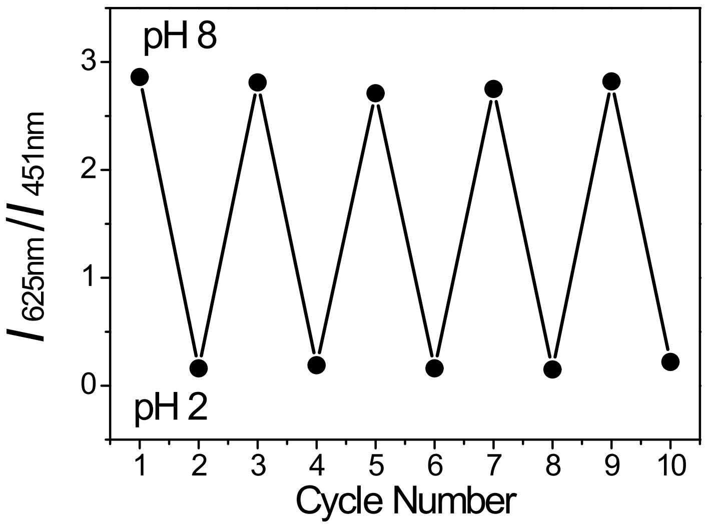


**Fig. S3** pH reversibility study of **S1** between pH 2 and 8.

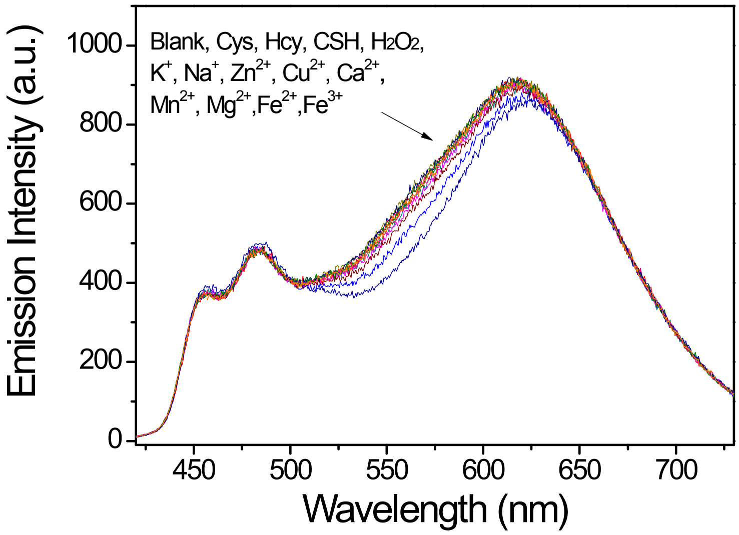


**Fig. S4** Phosphorescence spectra of **S1** ($2.0 \times 10^{-5}$ M) in the presence of $1.0 \times 10^{-4}$ M of oxidative-stress-associated redox chemicals and metal ions (Cys, Hcy, GSH, $H_2O_2$, $K^+$, $Na^+$, $Zn^{2+}$, $Cu^{2+}$, $Ca^{2+}$, $Mn^{2+}$, $Mg^{2+}$, $Fe^{2+}$ and $Fe^{3+}$) in $CH_3CN$/buffer (1 : 9, v : v).

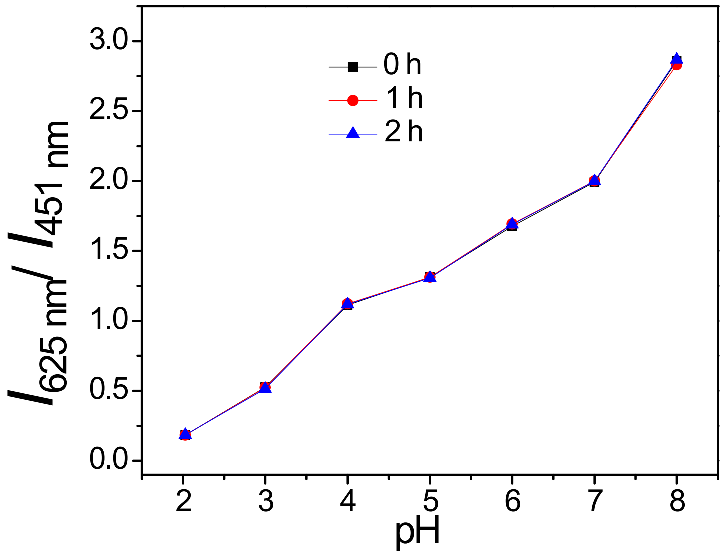


**Fig. S5** Relative ratio of phosphorescence intensity ($I_{625}$ nm/$I_{451}$ nm) changes of **S1** in the pH range of 2–8 at 37 °C.

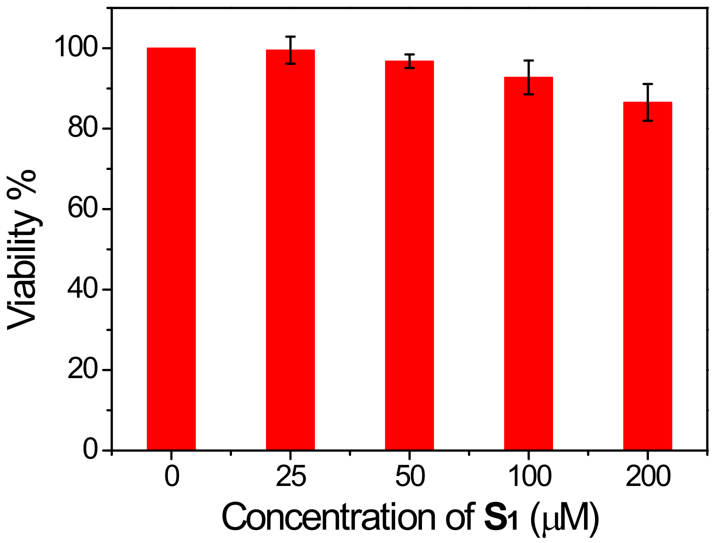


**Fig. S6** Cell viability values (%) assessed using an MTT test versus incubation concentrations of **S1**. HepG-2 cells were cultured in the presence of 0–200 μM **S1** at 37 $^{o}C$ for 24 h.

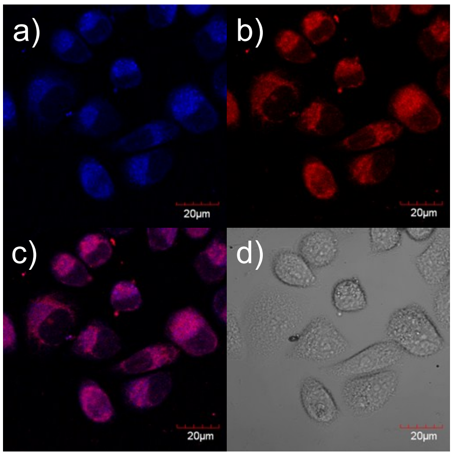


**Fig. S7** (a, b) Confocal luminescence, (c) overlay images and (d) bright-field of living HepG-2 cells. HepG-2 cells were incubated with 10 μM **S1** for 1 h at 37 °C.

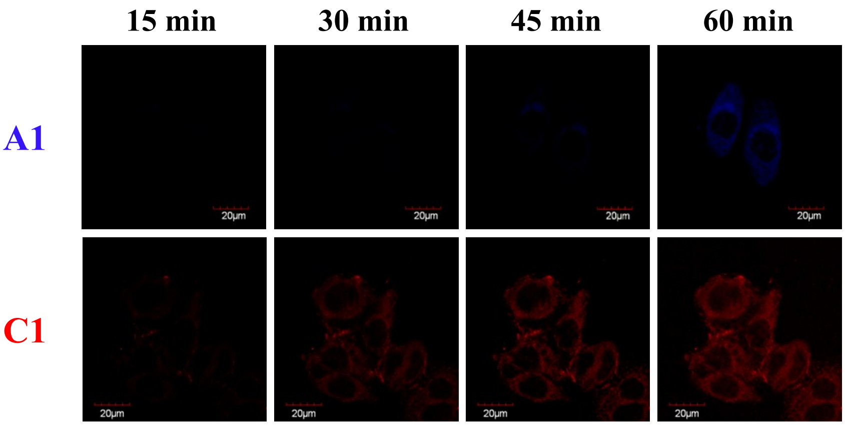


**Fig. S8** Real-time monitoring of live cells stained with **A1** and **C1**, respectively.

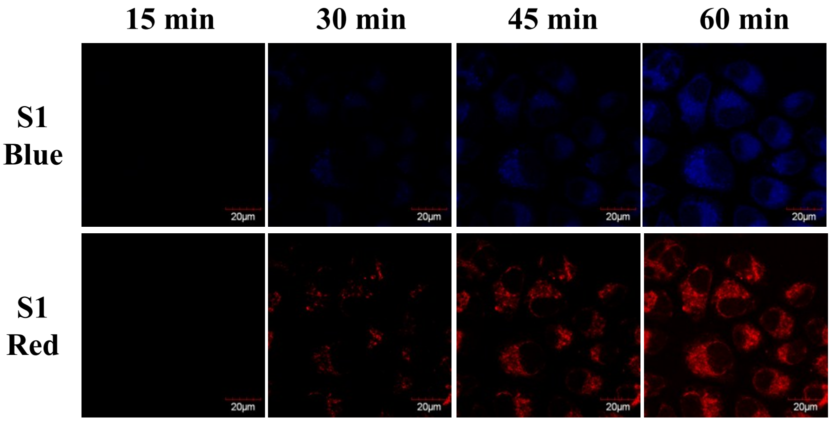


**Fig. S9** Real-time monitoring of live cells stained with **S1**.

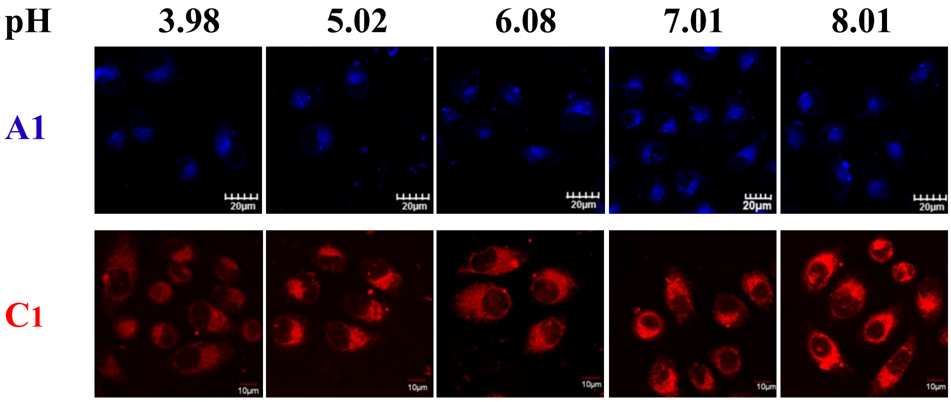


**Fig. S10** Phosphorescence images of **A1** and **C1** in HepG-2 cells clamped at pH 3.98, 5.02, 6.08, 7.01 and 8.0, respectively.

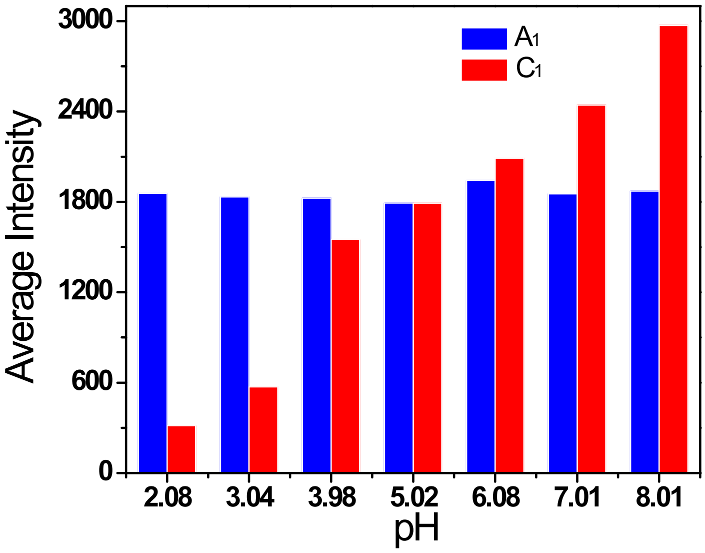


**Fig. S11** Average intracellular emission intensity of **A1** and **C1** at different pH values.

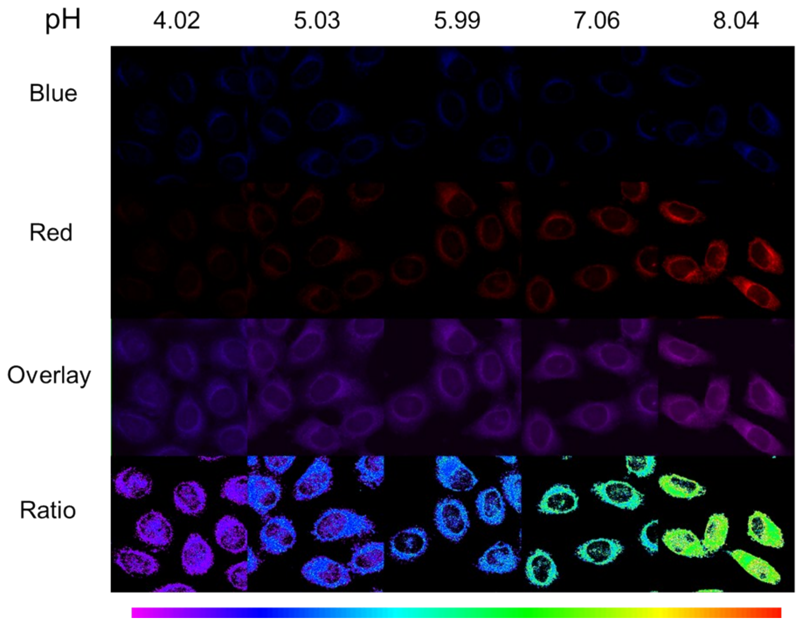


**Fig. S12** Phosphorescence images of **S1** (1 μM) in living cells clamped at pH 4.02, 5.03, 5.99, 7.06 and 8.04, respectively. The excitation wavelength was 405 nm and the images of the first row (blue channel) and second row (red channel) were collected in the ranges of 430–480 nm and 600–700 nm, respectively. Overlay images (third row) and ratio images obtained from the red and blue channels (fourth row).

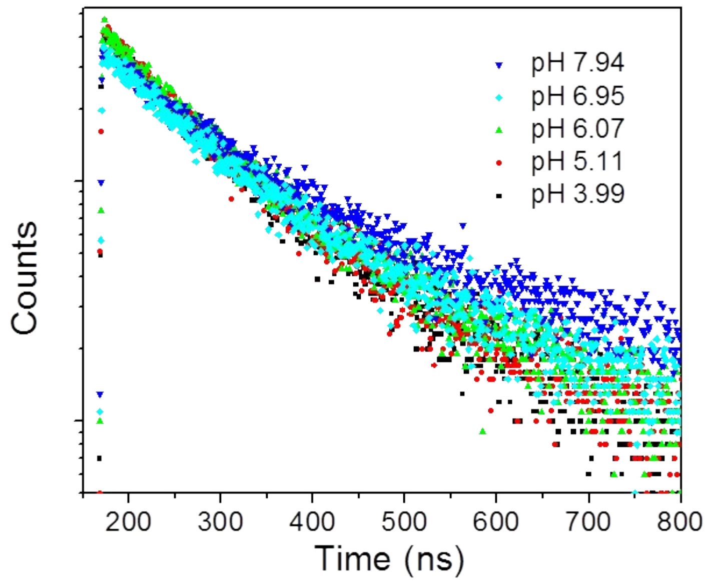


**Fig. S13** Phosphorescence decay curves of **S1** (10 μM) at 625 nm at different pH values.

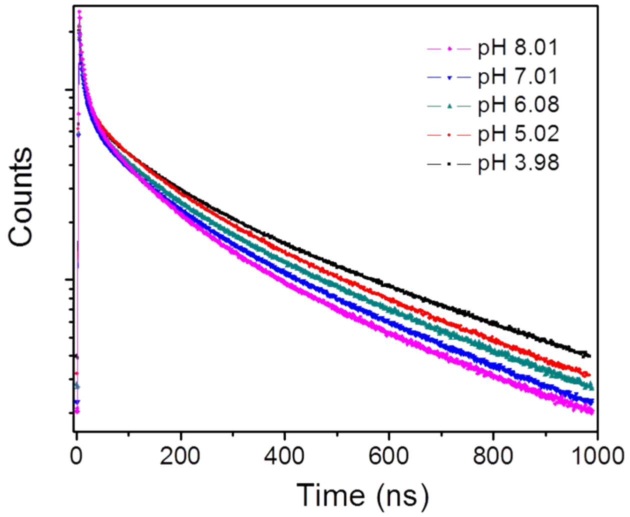


**Fig. S14** Phosphorescence decay curves in cells stained with **S1** (10 μM) at different pH values, obtained by confocal TCSPC-PLIM.

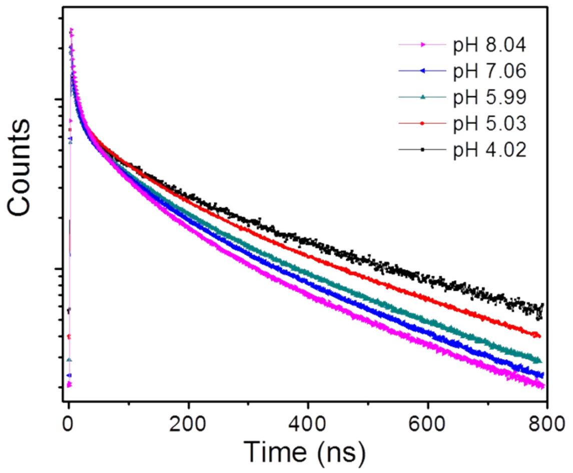


**Fig. S15** Phosphorescence decay curves of **S1** (10 μM) in RPMI 1640 at different pH values, obtained by confocal TCSPC-PLIM.

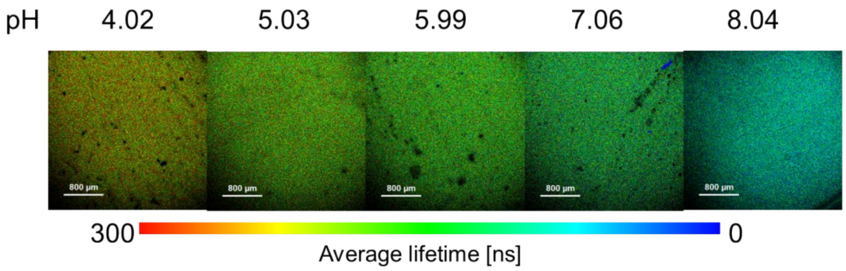


**Fig. S16** Phosphorescence lifetime images of **S1** (10 μM) in RPMI 1640 at different pH values.

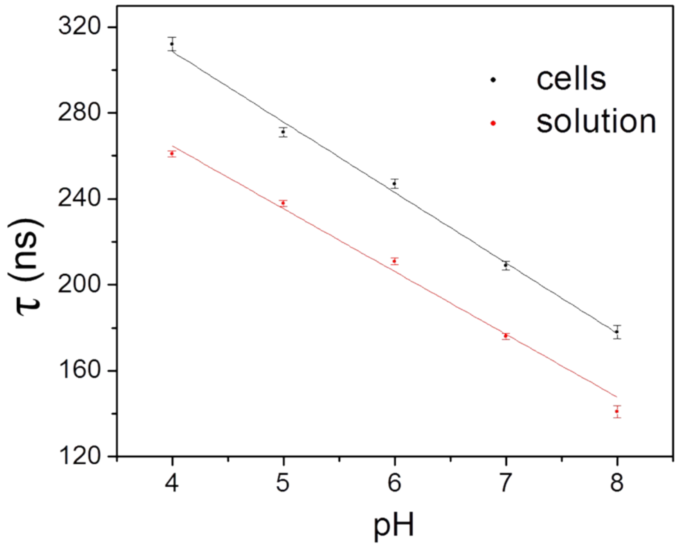


**Fig. S17** Emission lifetime changes of **S1** (10 μM) at different pH values in the cells and RPMI 1640, respectively, obtained by confocal TCSPC-PLIM.

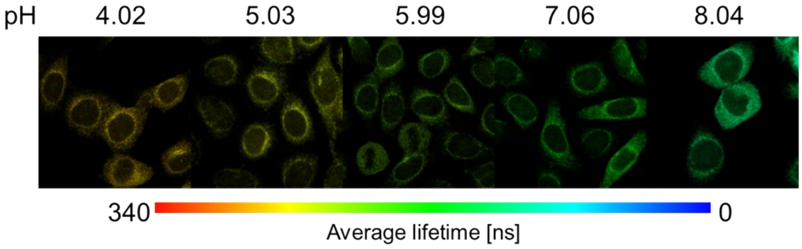


**Fig. S18** Phosphorescence lifetime images of **S1** (1 μM) in living cells at different pH values.

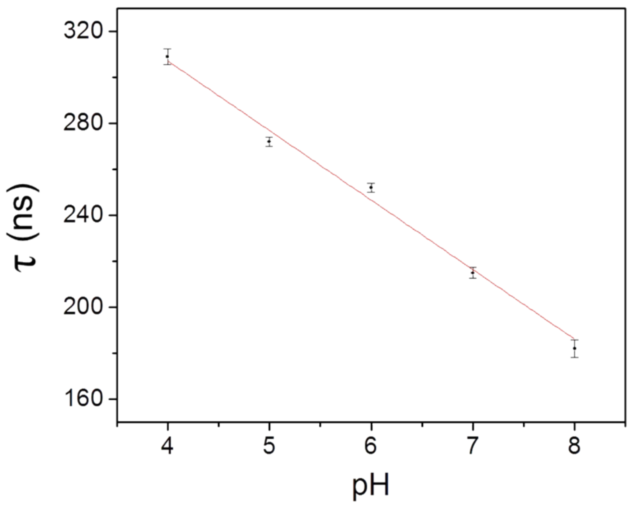


**Fig. S19** Emission lifetime changes of **S1** (1 μM) at different pH values in the cells, obtained by confocal TCSPC-PLIM.

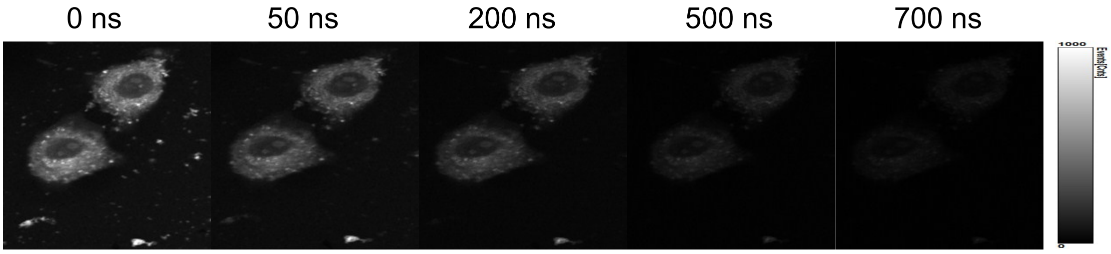


**Fig. S20** Phosphorescence intensity images collected at different time ranges (0-700 ns).

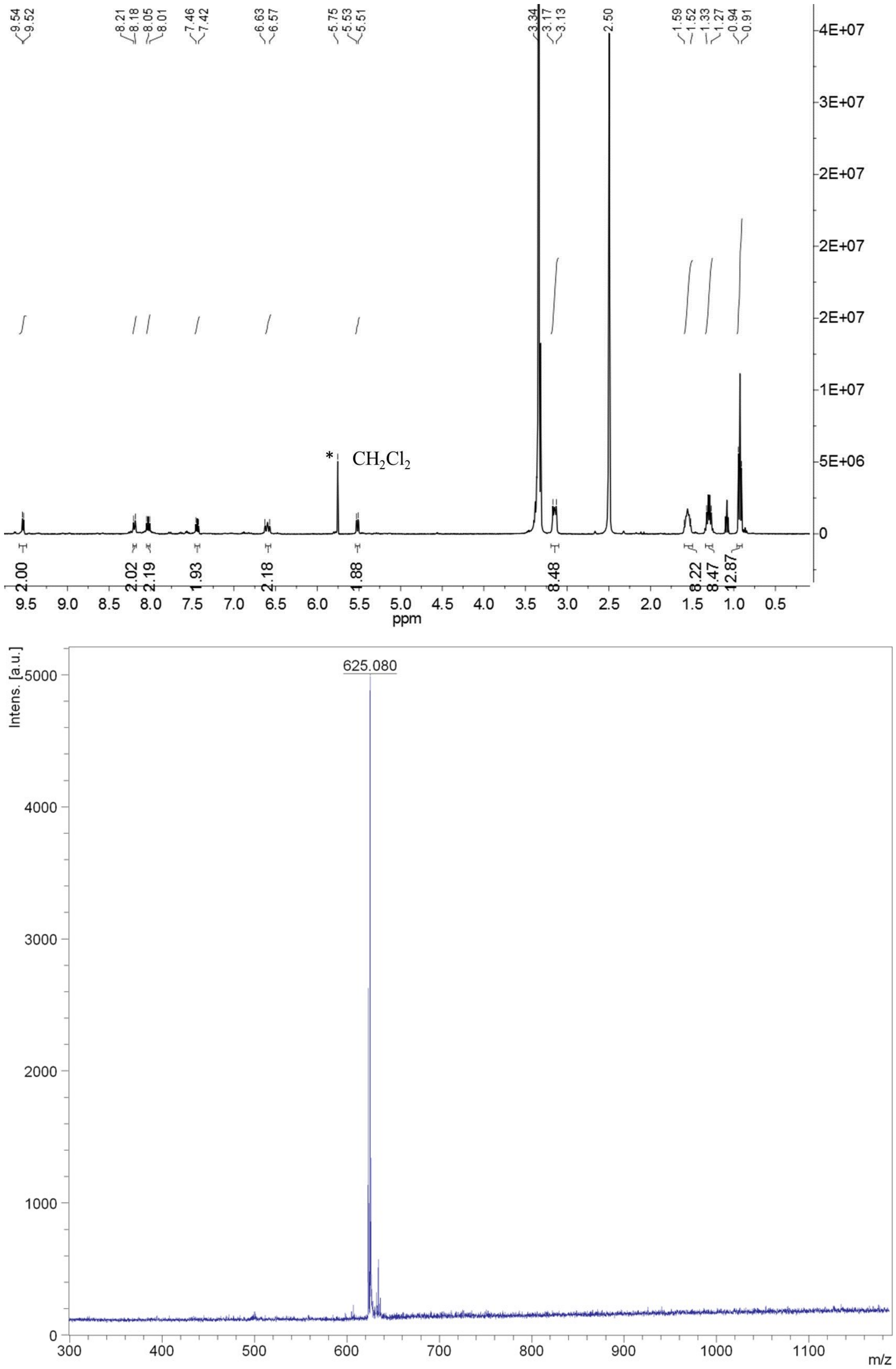


**Fig. S21** $^{1}$H NMR (400 MHz, DMSO-$d_6$) and mass spectra of **A1**.

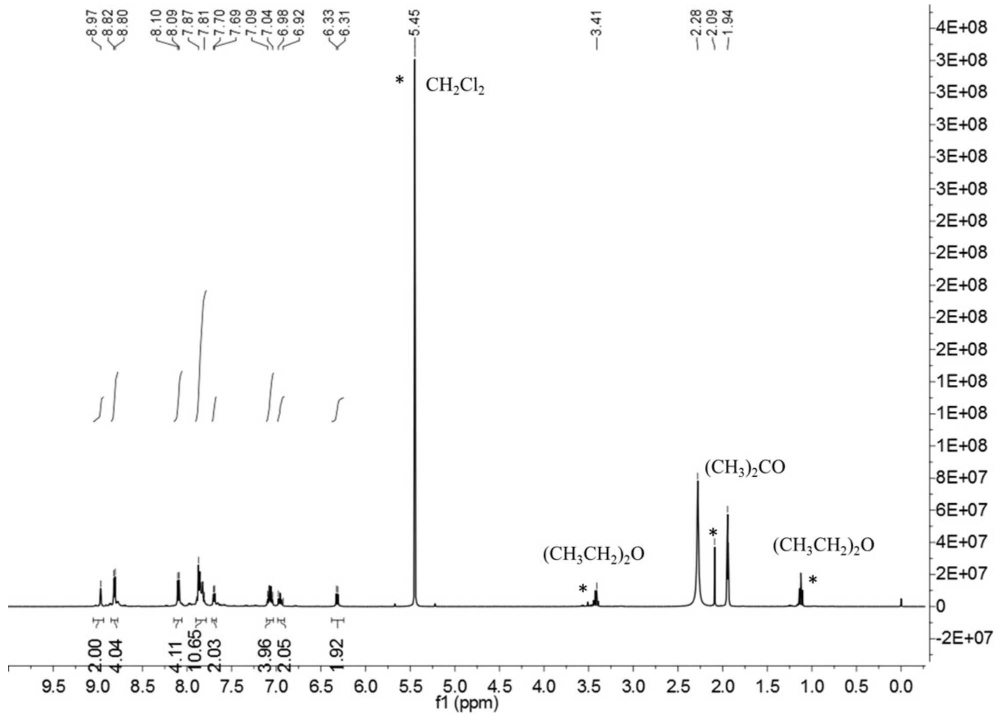


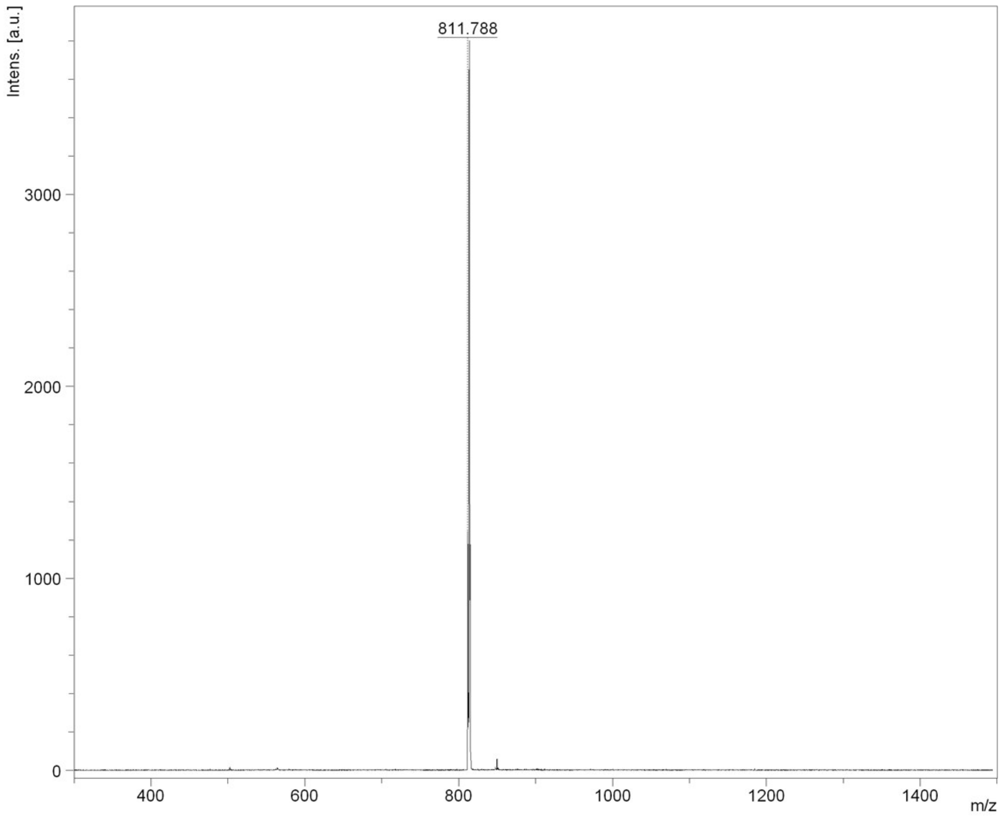


**Fig. S22** $^{1}H$ NMR (400 MHz, acetonitrile-$d_3$) and mass spectra of **C1**.

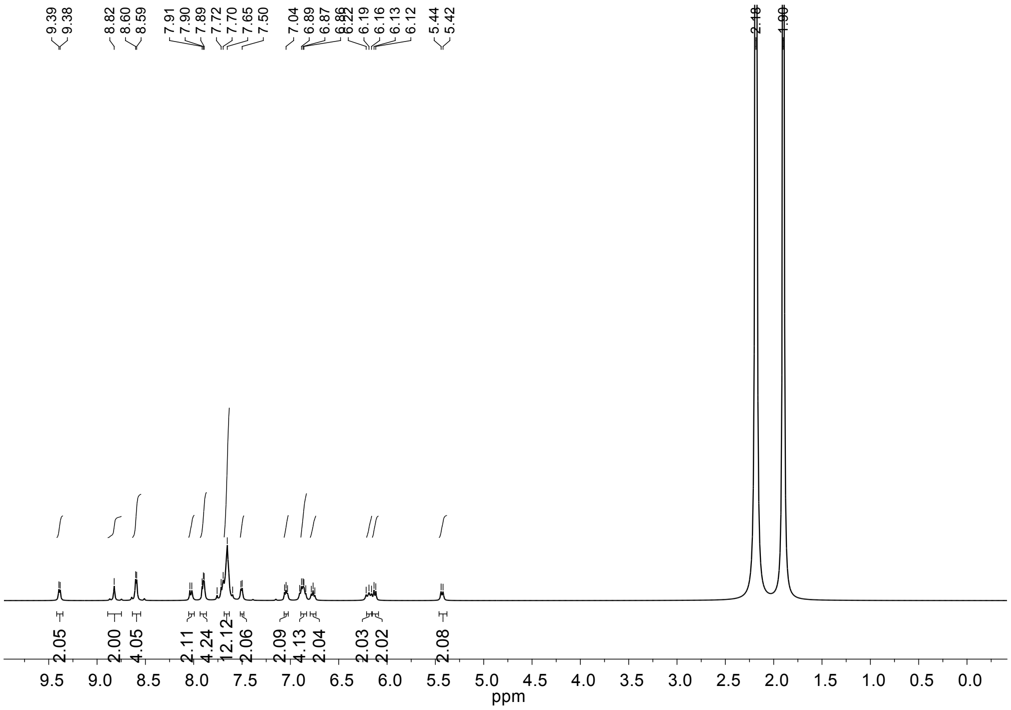

**Fig. S23** $^1$H NMR (400 MHz, acetonitrile-$d_3$) spectrum of **S1**.

**Table S1** Photophysical properties of complexes **A1, C1** and **S1**

| Complexes | Solvent | $\lambda_{abs}$, nm (logε) | $\lambda_{PL}$, nm | $\tau$, ns | $\Phi_{em}$ |
|---|---|---|---|---|---|
| **A1** | $CH_3CN$ | 251 (5.21)<br>293(4.83)<br>364(4.31) | 451, 475 | 1329 | 0.62 |
| **C1** | $CH_3CN$ | 249 (4.99)<br>291 (4.73)<br>386 (4.10) | 633 | 413 | 0.18 |
| **S1** | $CH_3CN$ | 249 (5.21)<br>289 (4.90)<br>364 (4.34) | 453, 480,<br>633 | 819/398 | 0.22 |
